# Facile Chemical Bath Synthesis of SnS Nanosheets and Their Ethanol Sensing Properties

**DOI:** 10.3390/s19112581

**Published:** 2019-06-06

**Authors:** Wei Shan, Zhengqian Fu, Mingsheng Ma, Zhifu Liu, Zhenggang Xue, Jiaqiang Xu, Faqiang Zhang, Yongxiang Li

**Affiliations:** 1 CAS Key Lab of Inorganic Functional Materials and Devices, Shanghai Institute of Ceramics, Chinese Academy of Sciences, Shanghai 200050, China; shanwei@student.sic.ac.cn (W.S.); fmail600@mail.sic.ac.cn (Z.F.); mamingsheng@mail.sic.ac.cn (M.M.); zhangfq@mail.sic.ac.cn (F.Z.); 2 Center of Materials Science and Optoelectronics Engineering, University of Chinese Academy of Sciences, Beijing 100049, China; 3 Department of Chemistry, Shanghai University, Shanghai 200444, China; xuezhenggang@163.com (Z.X.); xujiaqiang@shu.edu.cn (J.X.)

**Keywords:** gas sensors, SnS nanosheets, chemical bath synthesis, oleylamine, ethanol sensing

## Abstract

Tin(II) monosulfide (SnS) nanosheets were synthesized using SnCl_4_•5H_2_O and S powders as raw materials in the presence of H_2_O via a facile chemical bath method. Orthorhombic phase SnS nanosheets with a thickness of ~100 nm and lateral dimensions of 2~10 μm were obtained by controlling the synthesis parameters. The formation of a SnO_2_ intermediate is key to the valence reduction of Sn ions (from IV to II) and the formation of SnS. The gas sensors fabricated from SnS nanosheets exhibited an excellent response of 14.86 to 100 ppm ethanol vapor when operating at 160 °C, as well as fast response and recovery times of 23 s and 26 s, respectively. The sensors showed excellent selectivity for the detection of ethanol over acetone, methanol, and ammonia gases, which indicates the SnS nanosheets are promising for high-performance ethanol gas sensing applications.

## 1. Introduction

Ethanol (EtOH), a typical volatile organic compound (VOC), is a common raw material and has important applications in the chemical, biomedical, and food industries. However, it is volatile and flammable, and it can cause health problems such as headache and liver damage [1]. The detection of EtOH gas/vapor is therefore of particular importance. Nowadays, semiconductor gas sensors are most widely used due to their high sensitivity, ease of fabrication, simple structure, and low cost [2]. For example, SnO_2_ [3], ZnO [4], In_2_O_3_ [5], and WO_3_ [6] have been employed to fabricate ethanol gas sensors. However, most oxide semiconductor gas sensors tend to exhibit high operating temperatures [7,8,9] which increase the power consumption, accelerate the aging of gas-sensing materials, and reduce their service life. Recently, TiO_2_ and Fe_2_O_3_ have been employed to make gas sensors which can work at room temperature [10,11,12]. Bhowmik et al. made an EtOH sensor device based on the p–n homojunction of TiO_2_ nanostructures [10]; it offered a maximum response magnitude of ∼57% (toward ethanol) at 100 ppm with appreciably fast response and recovery times of ∼30 and ∼16 s, while the nano-heterostructure increased the difficulty of device fabrication. Bindra et al. fabricated multi-layered TiO_2_ nanotube-array-based highly sensitive room-temperature vapor sensors [11], but the response time was more than 82 s; Lin et al. made an EtOH gas sensor based on *γ*-Fe_2_O_3_ nanoparticles working at room temperature [12], but the response time was up to 533 s. In addition, most oxide semiconductor ethanol-gas-sensitive materials also have strong interference signals for other reducing VOCs, among which the most representative interference sources are acetone and methanol. At present, commercial EtOH sensors are composed of an array of gas-sensing materials with different selectivity, and the gas recognition and composition analysis are performed by pattern recognition technology [13,14], but these sensing arrays have complicated structures. So, it is still necessary to develop a gas-sensing material which has low operating temperature, high sensitivity, good selectivity, and a fast response time to EtOH gas.

Recently, flak-like sulfide materials have attracted great interest because of their excellent electrical, optical, and mechanical properties [15,16,17], as well as their planar crystal structure, large surface-to-volume ratio, and low electronic noise [18,19]. These properties make sulfide nanosheets very suitable for gas-sensing materials; this has not received the deserved attention. In most cases, sulfide-nanosheet-based gas sensors have a lower operating temperature than do metal oxide semiconductor materials [19,20,21]. This is mainly due to the S atoms exposed on the surface of sulfide nanosheets that have less electronegativity and more easily absorb oxygen in the air. At the same time, the narrower band gap of the sulfide material (<2.5 eV), compared with that of oxide semiconductor materials (~3.0 eV), can reduce the power consumption of gas sensors. Additionally, previous research based on first-principles calculations and experiments has shown that physisorption-based charge transfer functions more easily between the surface of flaky sulfide gas-sensitive materials and target gas molecules at lower temperatures, which may result in better gas sensing selectivity for a particular target gas [20,22,23].

As a new 2D material with a black phosphorene structure, SnS has an energy band gap of about 1.3 eV [24,25], which is suitable for a gas-sensing material. Recently, Rana et al. fabricated a gas sensor based on SnS nanoparticles [26] which had high sensitivity and a fast response time to EtOH gas. This confirmed that SnS is a very promising ethanol gas-sensing material. Three kinds of methods have been used to synthesize SnS nanomaterial: top-down exfoliation from bulk materials, chemical vapor deposition (CVD), and solution synthesis [27,28,29,30,31,32]. However, the nanosheets obtained by exfoliation methods from bulk materials are not controllable in terms of their uniformity, size, and thickness, which is not suitable for large-scale production. Although O’Brien et al. prepared SnS thin films via CVD [29], the complex precursors and strict synthesis conditions limited the application of this method. Herron et al. prepared SnS nanosheets by a solution method, but the particles were small and thick [30]. Deng et al. synthesized ultrathin single crystalline SnS in oleylamine (OAm) but with the addition of 1-dodecanethiol (DDT), which is a highly toxic pesticide [31]. Cui et al. prepared large-sized SnS thin crystals by solvothermal methods; however, the reaction pressure was up to 200 MPa, which is hard to achieve and dangerous in operation [32]. Therefore, there is still a significant need to explore a simple, efficient, and environmentally friendly process to grow large-sized SnS nanosheets.

In the present work, we report a facile chemical bath method to synthesize large-sized SnS nanosheets from cheap and environmentally benign chemicals. The gas sensing results of the synthesized SnS nanosheets show a high response, great selectivity, and fast response and recovery times towards EtOH gas.

## 2. Materials and Methods

### 2.1. SnS Nanosheet Synthesis and Characterization

**Figure 1:**
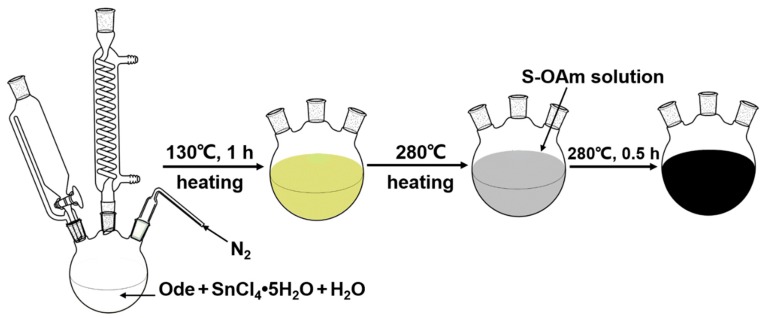
Diagram of the chemical bath synthesis process of SnS nanosheets.

Tin (IV) chloride (SnCl_4_•5H_2_O, >99.9%, Aladdin) and sulfur powder (5N, Aladdin) were used as the tin (Sn) and sulfide (S) precursors, respectively; oleylamine (OAm, >90.0%, Aladdin) was used as a surfactant; and octadecene (Ode, >90.0%, Aladdin) was the solvent. All the chemical reagents were used as received without further purification. Figure 1 shows a diagram of the synthesis process. A simplified Schlenk line was used to protect the reaction from oxygen/air, and the whole synthesis process should be performed under a persistent flow of high-purity N_2_ gas with constant stirring.

In a typical experiment, SnCl_4_•5H_2_O (1 mmol) was dissolved into distilled water (10 mL), then the above solution and Ode (20 mL) were added into a 50 mL three-neck flask. This mixed solution was degassed at 130 °C for 1 h to fully hydrolyze SnCl_4_•5H_2_O and evaporate excess water. Subsequently, the solution was heated to 280 °C within 15 min. The S-OAm solution (1 mmol of sulfur powder dispersed into 10 mL of OAm) was injected into the reaction system, and the reaction was maintained at 280 °C for 30 min. After cooling the solution to room temperature naturally, a gray-black product was collected and separated from the solution by centrifugation. The product was further washed three times using an ethanol and cyclohexane mixed solvent (1:1 volume ratio) and finally dispersed in cyclohexane. The morphology of the products was examined by scanning electron microscopy (SEM, JEOL S4800) equipped with an energy-dispersive X-ray (EDX) spectroscope and a transmission electron microscope (TEM, JEM-2100F, JEOL). Powder X-ray diffraction (XRD) patterns were taken on an X’Pert Pro MPD (Philips PAN analytical) with Cu Kα radiation at 45 kV and 40 mA. The XRD data were collected with the θ-2θ scanning scope starting from 2θ = 5° to 80° with a step of 6° per second by using Cu Kα radiation (1.5418 Å). The composition of the reaction liquid was characterized by Fourier transform infrared spectrometry (FT-IR, 8400 Shimadzu).

### 2.2. The Gas Sensing Test

**Figure 2:**
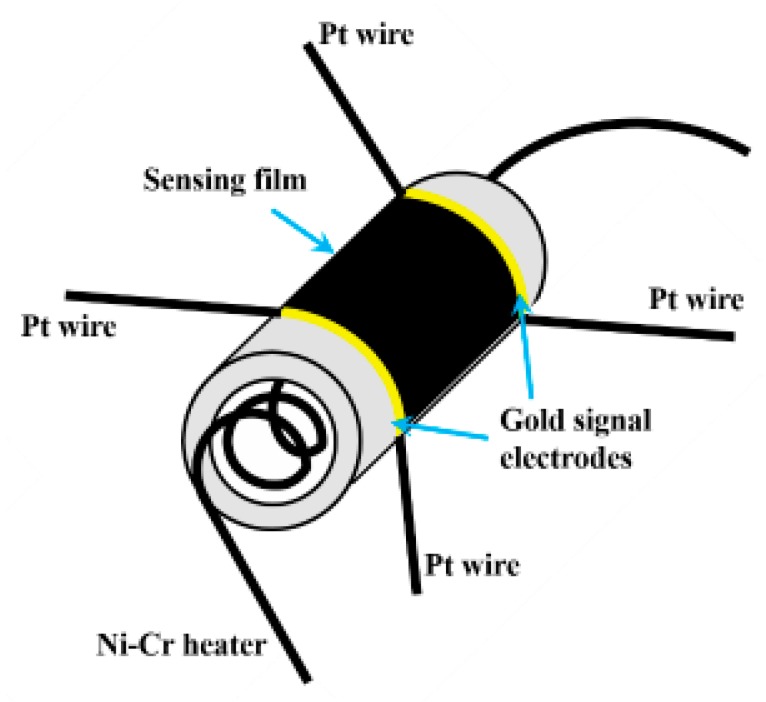
Schematic diagram of the device for gas sensing performance testing.

To make the gas sensing device, 0.1 g of as-prepared SnS nanosheets and 5 mL of cyclohexane were added into a centrifuge tube with 5 mL capacity. This mixture was ultrasonicated for 10 min to form a concentrated suspension. An alumina ceramic tube (Figure 2) with two ring-shaped gold electrodes and two platinum wires connected to each electrode were put into the suspension such that the surface of the tube was coated with SnS nanosheets and then dried in ambient air naturally. Repeating the above processes five times, a gas sensing test tube whose surface was completely and evenly covered by SnS nanosheets was prepared. Then, a Ni–Cr alloy filament was put into the ceramic tube as a micro heater. The operating temperature of the gas sensor is controlled by the voltage across the heating wire. The final gas sensing test unit was assembled by soldering the ceramic tube with SnS nanosheet sensing material and electrodes onto a connector base with six probes.

The gas sensing tests were carried out using a commercial HW-30A measurement system (Han Wei Electronics Co., Zhengzhou, China) using ambient air as the dilute and reference gas. For details on this test system, please see the Supplementary Materials. A hygrometer showed a humidity of 60% at room temperature (25 °C). The response time (t_res_) or recovery time (t_rec_) is expressed as the time taken for the sensor output to reach 90% of its saturation after the test gas is applied or deactivated in a function step. The response *S* was defined as

*S* = *R_g_*/*R_a_*, (1)

where *R_a_* and *R_g_* are the steady-state resistances in air and the testing gas, respectively.

## 3. Results and Discussion

### 3.1. Microstructural Characterizations of the SnS Nanosheets

**Figure 3:**
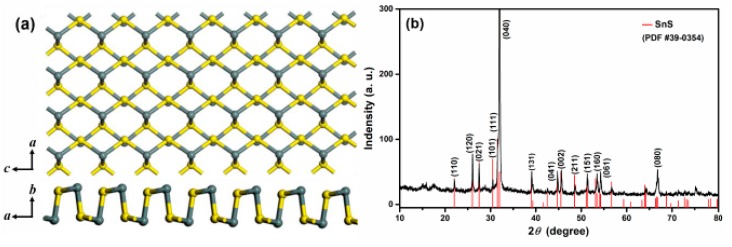
(**a**) Crystal structure diagram of orthorhombic phase SnS. (**b**) XRD pattern of typical SnS nanosheets.

**Figure 4:**
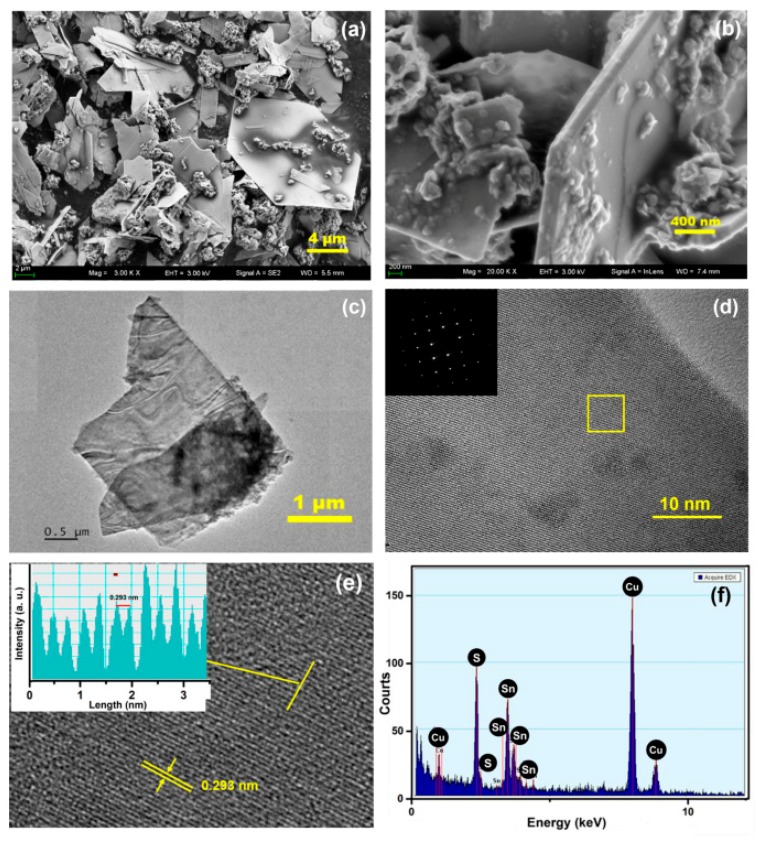
(**a**) and (**b**) SEM images of SnS nanosheets. (**c**) TEM image of SnS nanosheets. (**d**) and (**e**) HRTEM images of SnS nanosheets. (**f**) The EDX spectrum of SnS nanosheets.

Figure 3a shows the crystal structure diagram of SnS. Figure 3b is the XRD pattern of as-prepared typical SnS nanosheets, which share the same structure as orthorhombic phase SnS (JCPDS No. 39-0354) with a space group of Pbnm(62). All sharp peaks match the JCPDS card, and no impurities were identified. In this crystal structure, S atoms and Sn atoms in the same two-atom-thick layer are alternately arranged in a zigzag (Figure 3a), and two adjacent layers are held together by van der Waals forces [33]. Each unit cell contains two of these two-atom-thick layers along the *b* axis. The primary diffraction peak at the 2*θ* value of 31.97°, ascribed to the (040) plane, is consistent with the SEM and TEM images in Figure 4. The SEM images (Figure 4a,b) display the morphology of typical SnS nanosheets. Most as-prepared SnS particles are sheet-like with lateral sizes of 5~20 μm and a thickness of about 100 nm. Figure 4c shows a TEM image of a monodisperse SnS nanosheet, and Figure 4d is a high-resolution TEM (HRTEM) picture of the square area in Figure 4c. The corresponding selected area electron diffraction (SAED) pattern reveals the single-crystalline structure of orthorhombic phase SnS nanosheets with the extending direction parallel to the (040) plane. Figure 4e shows an HRTEM image of the region circled by the frame in Figure 4d, and the inset is a profile intensity image along the line, in which a lattice fringe spacing of 0.293 nm is identified. It should correspond to the ($\bar{1}01$) or (101) lattice planes of orthorhombic phase SnS. EDX analysis was performed and strong signals from Sn and S were detected, as shown in Figure 4f. The peaks related to Cu came from the copper mesh substrate used in the TEM analysis. The calculated composition ratio was 1.03(Sn):0.97(S), with a slight sulfur deficiency compared to the stoichiometric SnS. It is important that the deficient S atoms make the as-prepared SnS nanosheets exhibit n-type semiconducting characteristics. The high aspect ratio and large area of the high-quality (040) surface endue it with promising applications like catalysis and gas sensing.

### 3.2. Synthesis Process and Mechanism

**Figure 5:**
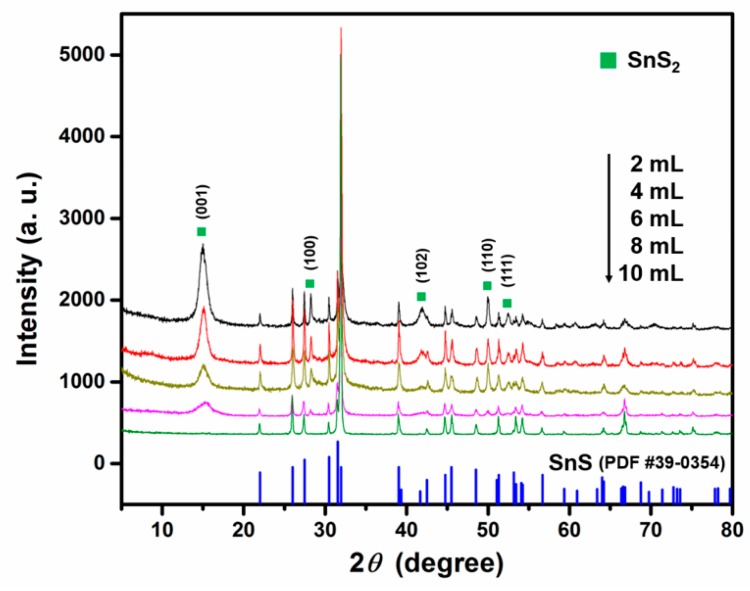
XRD patterns of samples synthesized with different volumes of water.

To understand the reaction process, especially the valence conversion of Sn ions from +4 to +2, we conducted a series of characterizations to different time-dependent intermediate products. Firstly, a series of samples were prepared with different volumes of water (2 mL, 4 mL, 6 mL, 8 mL, and typical 10 mL) while the other conditions remained unchanged. Figure 5 shows the XRD patterns of these samples. When there is no water addition during the reaction, the main products are hexagonal crystal SnS_2_ flakes. However, the main product changed to orthorhombic crystal SnS when even a small amount of water was introduced into the reaction system. With an increasing amount of water, the yield of SnS in the product increases. Pure SnS nanosheets were obtained when 10 mL water was added in the reaction. This demonstrates that the added water is not only a solvent but also participates in the chemical reaction.

**Figure 6:**
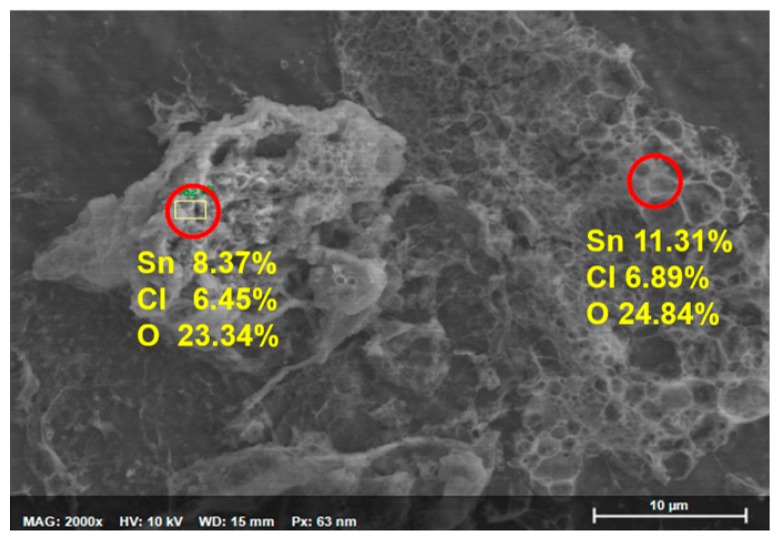
SEM image of SnCl_4_ hydrolyzates and the element composition of the circled area measured by EDX.

The intermediate product was further analyzed in order to understand the function of water in the reaction process. Figure 6 shows an SEM image of a sample obtained by heating the reactants at 130 °C for 1 h and then by centrifugation. It is apparently not a crystallized substance. The EDS analysis results indicate that it contains Sn, O, and Cl elements with an atom ratio of about 1(Sn):3(O):1(Cl). In addition, we passed the effluent gas through a flask with deionized water. After the reaction, the testing water became acidic, which indicates the formation of hydrochloric acid (HCl). It is possible that SnCl_4_ was hydrolyzed during the heat preservation process following the chemical reaction

(2) $${\mathrm{SnCl}}_{4}\;+\;H_{2}O\;\overset{\Delta }{\Rightarrow }\;\mathrm{Sn}(\mathrm{OH}{)}_{3}\mathrm{Cl}\;+\;\mathrm{HCl}\;↑.$$

**Figure 7:**
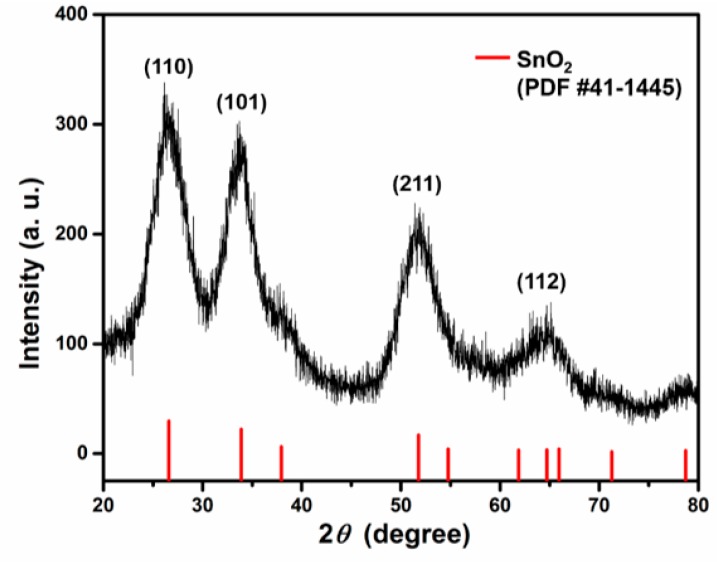
XRD pattern of the intermediate product before S-OAm reactant was added.

Moreover, the reaction was stopped when the reaction temperature had just reached 280 °C and the S-OAm solution had not been injected into the reaction flask. The reaction mixture was centrifuged and washed with EtOH. Finally, reddish-black powders were obtained. The XRD pattern shown in Figure 7 indicates that the powder is a tetragonal crystal of SnO_2_ (JCPDS No. 41-1445) with a low degree of crystallinity. It is possible that the intermediate compound Sn(OH)_3_Cl was decomposed to form SnO_2_ at an elevated temperature (280 °C). After injection of the S-OAm solution into the reaction system, both reactions (4) and (5) may take place [34]. The SnS nanosheets can be formed following reaction (6).

Sn(OH)_3_Cl → SnO_2_ + HCl + H_2_O (3)

(4) $$\mathrm{R-}{\mathrm{CH}}_{2}{\mathrm{-NH}}_{2}\;+\;{\mathrm{SnO}}_{2}\;\overset{\Delta }{\Rightarrow }\;\mathrm{R-}\mathrm{CO-}{\mathrm{NH}}_{2}\;+\;\mathrm{SnO}\;+\;H_{2}O$$

(5) $$\mathrm{R-}{\mathrm{CH}}_{2}{\mathrm{-NH}}_{2}\;+\;S_{x}\;\overset{\Delta }{\Rightarrow }\;\mathrm{R-}\mathrm{CS-}{\mathrm{NH}}_{2}\;+\;H_{2}S$$

(6) $$\mathrm{SnO}+H_{2}S\;\overset{\Delta }{\Rightarrow }\;\mathrm{SnS}\;+\;H_{2}O$$

**Figure 8:**
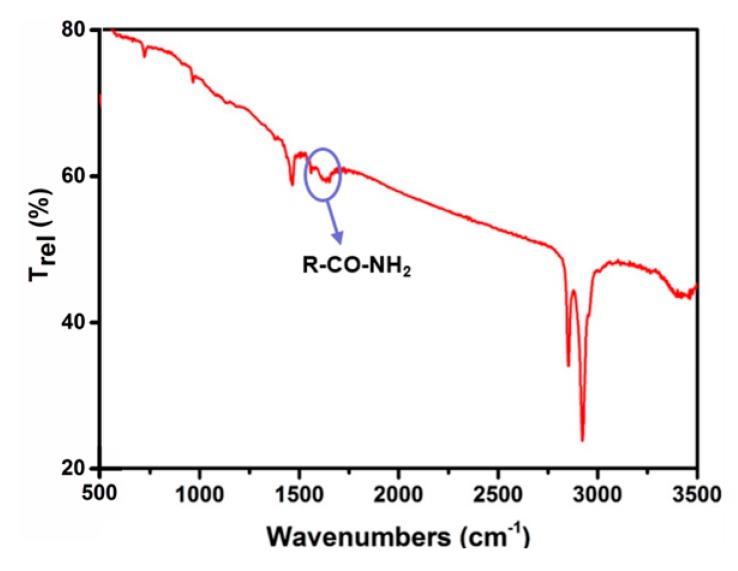
FI-IR spectrum of the final reaction solution.

In order to verify this assumption, the suspension was allowed to stand for 6 h after completion of the reaction, and then the supernatant liquid was taken for FT-IR analysis. The FTIR spectrum is shown in Figure 8. The obvious peaks at 1560 and 1647 cm^−1^ are attributed to the R-CO–NH_2_ stretch of amide, which confirms the formation of amide during the chemical bath reaction [35]. As discussed above, it could be concluded that reactions (2)–(4) are the key to the valence conversion of the Sn ion from +4 to +2.

### 3.3. Gas Sensing Properties

**Figure 9:**
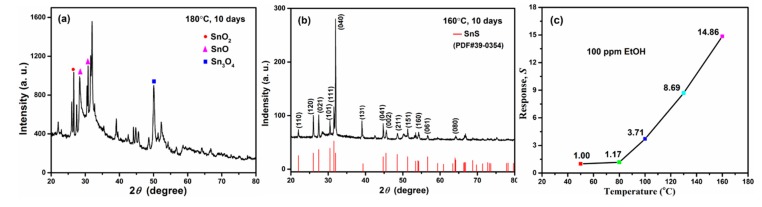
(**a**) XRD pattern of SnS samples which underwent heat treatment at 180 °C for 10 days in air. (**b**) XRD pattern of SnS samples which underwent heat treatment at 160 °C for 10 days in air. (**c**) The response *S* of sensors made of SnS nanosheets in the presence of 100 ppm of EtOH gas operating at different temperatures.

A large number of studies have demonstrated that the operating temperature of a gas sensor is the most influential factor which affects the gas response of semiconductor sensors [36]. It is known that sulfides are unstable in air at high temperatures, so we examined the temperature stability of the as-prepared SnS nanosheets by heating the samples to different temperatures. With the help of XRD characterization, we could clearly observe the transition from orthorhombic phase SnS to SnO_x_ after heating the nanosheets to 180 °C in air for 10 days, but this phenomenon did not appear with the same process at 160 °C, as shown in Figure 9a,b. Therefore, we tested the gas sensing response of SnS nanosheets to 100 ppm EtOH at operating temperatures from room temperature to 160 °C, and the results are shown in Figure 9c. At room temperature, there is no acceptable signal because the resistance of the sensing device is too big for the measurement range of the instrument. With increasing operating temperature, the response is enhanced, suggesting that increased temperature facilitates the adsorption of EtOH gas molecules onto the surface of SnS nanosheets and promotes the charge transfer [17]. This positive correlation between response *S* and operating temperature is valid until the temperature of 160 °C, at which the response *S* is 14.86. At present, some of the conversional metal oxide semiconductor (ZnO, WO_3_, etc.) gas sensors work at over 250 °C [1,37]. The low operating temperature of the SnS nanosheet gas sensor may be due to the following reasons. On one hand, the electronegativity of the S atom is lower than that of the O atom. Thus, compared with the surface of metal oxides, oxygen molecules are more easily adsorbed on the surface of tin sulfides and then capture electrons from the conduction band to form oxygen ions; this makes sensing signals based on chemical adsorption easy to produce. On the other hand, the physisorption-based charge transfer also contributes significantly to the gas sensing properties of flaky sulfide materials, which can work at lower temperatures [18,38].

**Figure 10:**
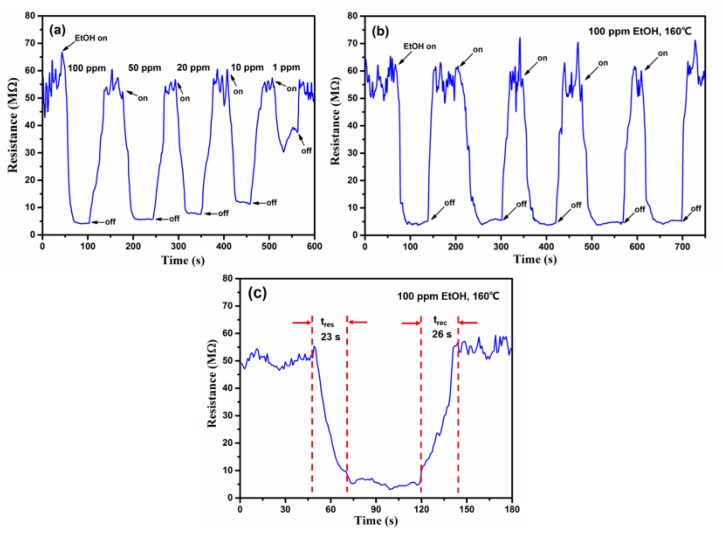
Gas sensing performance of SnS nanosheets: (**a**) Dynamic sensing performance of SnS nanosheets toward EtOH gas at concentrations ranging from 1 ppm to 100 ppm at 160 °C. (**b**) Five reversible test cycles of SnS nanosheets toward 100 ppm EtOH at 160 °C. (**c**) A single cycle test of SnS nanosheets after exposure to 100 ppm EtOH at 160 °C.

Figure 10a shows the dynamic response–recovery curves of the sensors to EtOH gas with concentrations ranging from 1 ppm to 100 ppm at 160 °C. Resistances of 35.7, 11.9, 7.9, 5.6, and 3.7 MΩ were recorded at EtOH gas concentrations of 1, 10, 20, 50, and 100 ppm, respectively. With decreasing EtOH concentration, the response signal is getting weaker and weaker. This is due to fewer ethanol molecules being adsorbed on the surface of the sensing material, and the resistance of the SnS nanosheets changes less. When the concentration of EtOH gas decreases to 1 ppm, the gas sensor still shows an obvious response *S* of 1.54, revealing that the resistance of the SnS nanosheets is very sensitive to EtOH vapor. The relationship between the response *S* and concentrations of NO_2_ is shown in the Supplementary Materials.

In order to study their reversibility characteristics, the sensing devices were exposed to 100 ppm of EtOH and air alternately for five or more cycles. It can be seen in Figure 10b that the resistance of the sensor in 100 ppm EtOH gas was steady and could return to the no-load level after each desorption. This result confirms that there is a minuscule amount of residual gas molecules during desorption process, and the SnS nanosheets gas sensors have a good performance in reversible cycle tests. The response and recovery times of the sensors to 100 ppm EtOH at 160 °C are shown in Figure 10c. As can be seen, the response and recovery times are 23 s and 26 s, respectively. These results are much better than those of most EtOH gas-sensing materials reported previously, such as Bi_2_S_3_ nanowires [39], CuxO films [40], WO_3_ microflowers [1], and V_2_O_5_ nanobelts [41]. 

**Figure 11:**
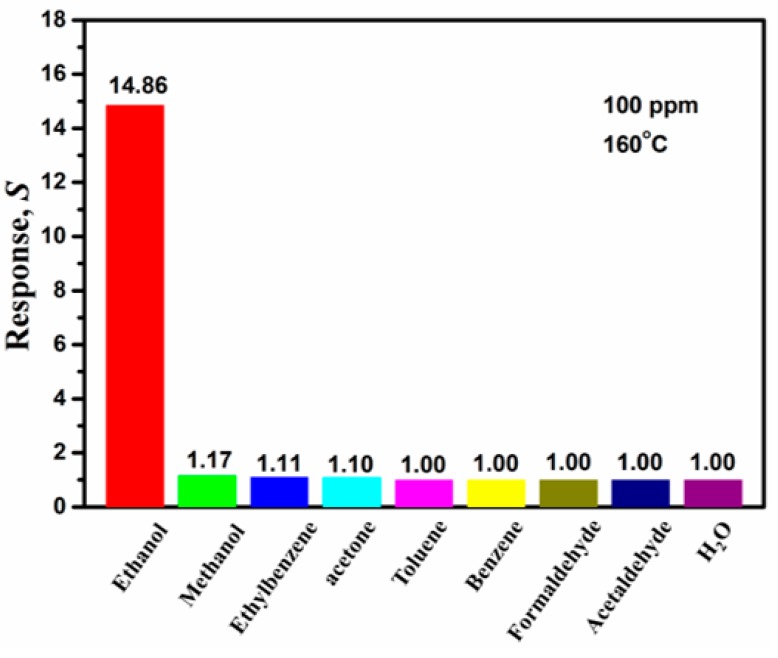
The response *S* towards various test gases at 100 ppm of SnS nanosheets at an operating temperature of 160 °C.

Figure 11 shows the response *S* of SnS-nanosheet-based sensors to 100 ppm of different gases at 160 °C. Compared to EtOH, other gases (including acetone and methanol gases, which are the most common sources of signal interference) have little response with the SnS-nanosheet-based gas sensor. In this work, the response *S* to EtOH is more than 10 times larger than those to the other gases. This indicates the SnS-nanosheet-based sensors exhibit a superior selectivity to EtOH gas. Ethanol gas is considered a representative of the reducing organic gases. According to previous studies, for volatile organic compounds (VOCs), the optimal operating temperatures depend on their molecular orbital levels [40]. When the lowest unoccupied molecular orbital (LUMO) level of the gas is low, the energy required for the gas sensing reaction is reduced. Since the LUMO level of ethanol (0.12572 eV) is lower than those of methanol (0.19728 eV) and acetone (0.20525 eV), electrons in the ethanol molecule are more prone to transfer than those in methanol and acetone molecules at low temperature [42,43]. In addition, physisorption-based charge transfer may play an important role in the gas sensing process. In this case, the more the electron cloud density shifts, the higher the molecule–surface binding energy becomes; this is reflected in the macro phenomenon in which the material resistance changes more and the gas sensing signal is stronger. Therefore, we speculate that the SnS nanosheets we prepared have higher binding energy to ethanol molecules than other tested organic molecules, especially methanol and acetone. This requires further experimentation or calculation to verify. As shown above, the SnS-nanosheet-based gas sensor in this study showed excellent selectivity to ethanol molecules.

## 4. Conclusions

In this work, SnS nanosheets were prepared using a simple solution reaction method. Orthorhombic phase SnS nanosheets with a thickness of ~100 nm and lateral dimensions of about 2~10 μm were obtained by controlling the synthesis parameters. The formation of a SnO_2_ intermediate is key to the valence reduction of Sn (from IV to II) and the formation of SnS nanosheets. The gas sensing properties of the SnS nanosheets were investigated at different operating temperatures. The SnS-nanosheet-based gas sensors exhibited excellent properties in sensing EtOH gas with a maximum response *S* of 14.86 at 160 °C, and a response time of 23 s and recovery time of 26 s. The response factor to EtOH is more than 10 times those to other VOCs. This study demonstrates the potential of SnS nanosheets for EtOH gas sensing applications.

## Supplementary Materials

The following are available online at https://www.mdpi.com/1424-8220/19/11/2581/s1, Figure S1: Photo of (a) the test circuit board and (b) HW-30A gas-sensing test system, Figure S2. Relationship between response S and concentrations of NO2 at 110 °C.
