# Supplementary material for: Facile Chemical Bath Synthesis of SnS Nanosheets and Their Ethanol Sensing Properties
Source: Sensors (Basel). 2019 Jun 6;19(11):2581. doi: 10.3390/s19112581 (PMC6603679; doi:10.3390/s19112581)

Facile Chemical Bath Synthesis of SnS Nanosheets and Their Ethanol Sensing Properties

Supporting Information

1. *HW-30A Gas-Sensing Test System*

We soldered the ceramic tube coated with SnS nanosheets to the gas sensor base, which was on the test circuit board, and then inserted the test circuit board into the gas sensing device. The test chamber had a volume of 20 L with a heating plate and two fans. When testing the gas-sensing response for a certain concentration of ethanol gas, we injected liquid EtOH onto the heating plate through a small hole in the glass cover. The EtOH liquid then evaporated quickly, and the fan kept the gas in the chamber flowing. The EtOH vapor rapidly diffused throughout the chamber to reach the test concentration.

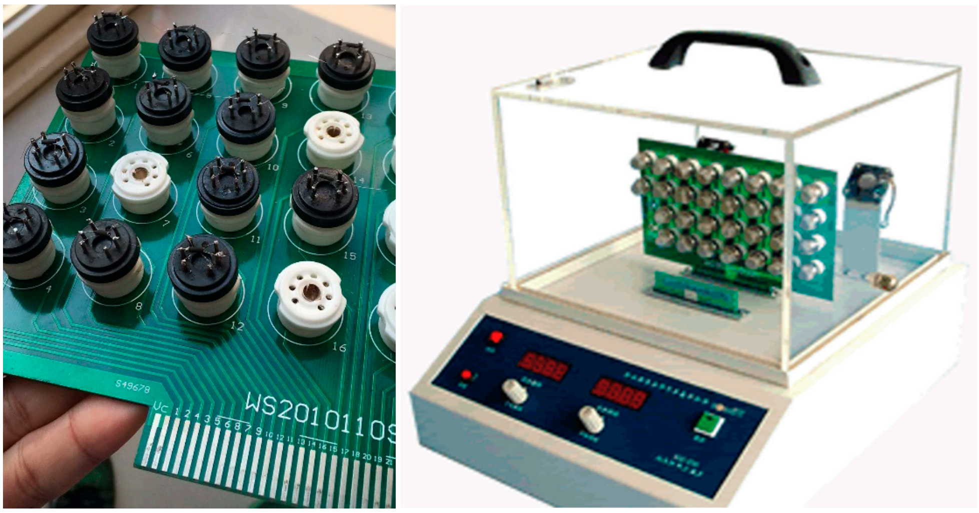

Figure S1. Photo of (**a**) the test circuit board and (**b**) the HW-30A gas-sensing test system.

2. *Relationship between Response* S *and the Concentration of* NO_2

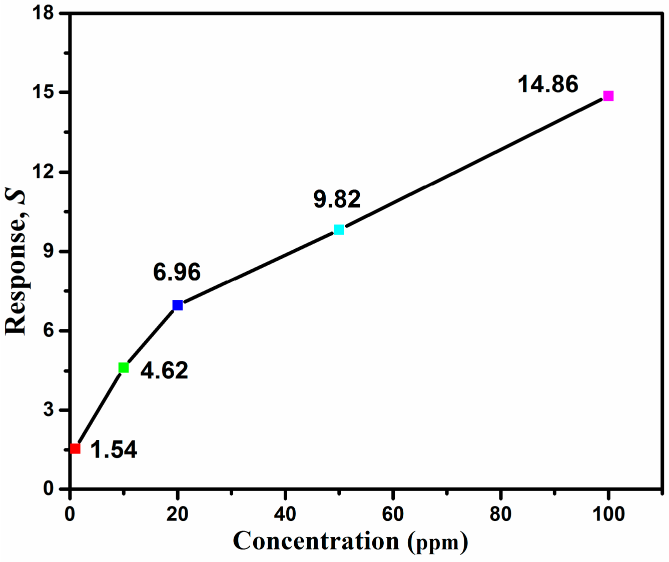

Supplement: Supplementary file 1 [file sensors-19-02581-s001.pdf]
